# Evolutionary Adaptation of Protein Turnover in White Muscle of Stenothermal Antarctic Fish: Elevated Cold Compensation at Reduced Thermal Responsiveness

**DOI:** 10.3390/biom13101507

**Published:** 2023-10-11

**Authors:** Nina Krebs, Christian Bock, Jan Tebben, Felix C. Mark, Magnus Lucassen, Gisela Lannig, Hans-Otto Pörtner

**Affiliations:** 1 Department of Integrative Ecophysiology, Alfred Wegener Institute, Helmholtz Centre for Polar and Marine Research, Am Handelshafen 12, 27570 Bremerhaven, Germany; christian.bock@awi.de (C.B.); felix.christopher.mark@awi.de (F.C.M.); magnus.lucassen@awi.de (M.L.); gisela.lannig@awi.de (G.L.); 2 Department of Ecological Chemistry, Alfred Wegener Institute, Helmholtz Centre for Polar and Marine Research, Am Handelshafen 12, 27570 Bremerhaven, Germany; jan.tebben@awi.de

**Keywords:** protein synthesis rate, protein degradation, polar fish, NMR, metabolic profiling, 13C-phenylalanine, non-radioactive, fish physiology, acute warming

## Abstract

Protein turnover is highly energy consuming and overall relates to an organism’s growth performance varying largely between species, e.g., due to pre-adaptation to environmental characteristics such as temperature. Here, we determined protein synthesis rates and capacity of protein degradation in white muscle of the cold stenothermal Antarctic eelpout (*Pachycara brachycephalum*) and its closely related temperate counterpart, the eurythermal common eelpout (*Zoarces viviparus*). Both species were exposed to acute warming (*P. brachycephalum*, 0 °C + 2 °C day^−1^; *Z. viviparus*, 4 °C + 3 °C day^−1^). The *in vivo* protein synthesis rate (Ks) was monitored after injection of ^13^C-phenylalanine, and protein degradation capacity was quantified by measuring the activity of cathepsin D *in vitro*. Untargeted metabolic profiling by nuclear magnetic resonance (NMR) spectroscopy was used to identify the metabolic processes involved. Independent of temperature, the protein synthesis rate was higher in *P. brachycephalum* (Ks = 0.38–0.614 % day^−1^) than in *Z. viviparus* (Ks= 0.148–0.379% day^−1^). Whereas protein synthesis remained unaffected by temperature in the Antarctic species, protein synthesis in *Z. viviparus* increased to near the thermal optimum (16 °C) and tended to fall at higher temperatures. Most strikingly, capacities for protein degradation were about ten times higher in the Antarctic compared to the temperate species. These differences are mirrored in the metabolic profiles, with significantly higher levels of complex and essential amino acids in the free cytosolic pool of the Antarctic congener. Together, the results clearly indicate a highly cold-compensated protein turnover in the Antarctic eelpout compared to its temperate confamilial. Constant versus variable environments are mirrored in rigid versus plastic functional responses of the protein synthesis machinery.

## 1. Introduction

Temperature strongly influences the performance of ectotherms such as in foraging, reproduction and growth. The temperature optimum of fish growth is not only species-specific and dependent on the temperature regime of its environment, it can also vary within species depending on season, life stage and habitat characteristics [1]. Accordingly, the range of temperatures that fish can thrive in, also known as the width of the thermal window, differs between species, life stages, regions and seasons [1,2,3]. Stenothermal organisms (e.g., polar and tropical fish) are well adapted to narrow temperature windows, whereas eurythermal organisms (e.g., fish from temperate zones) have a much wider thermal range and are more tolerant to fluctuations in temperature [4]. Polar stenothermal fish are able to maintain all life functions at temperatures constantly below 0 °C. Some functions such as growth are even slower in Antarctic ectotherms than would be expected on the basis of the Q_10_ relationship [5,6]. It is assumed that slow growth is caused by the constantly low temperatures (e.g., seasonal temperature range of Ryders Bay: −1.81–1.7 °C [7]) and a limited food supply due to seasonal changes [5,8,9]. Mechanism-based explanations include thermal tradeoffs between energy budget components such as ventilation and circulatory capacity, muscular activity and cellular and mitochondrial energy costs [10]. Overall, the mechanisms that influence thermal performance and especially the growth of polar ectotherms deserve further study for a deeper understanding of how temperature shapes the functioning of stenothermal fishes as opposed to eurythermal fishes [11].

Growth performance is defined as weight gain per day, which is the result of many different processes, usually measured over a long period of time (weeks to months) particularly in slow-growing Antarctic fish [12,13,14]. A highly important contributor to growth is protein synthesis. It has been suggested that protein homeostasis is a crucial factor limiting thermal performance in Antarctic organisms [6]. The protein synthesis rate can be measured in the whole organism (e.g., [15,16]) or, especially in fish, it can be approximated by studying protein synthesis in white muscle which contributes up to 79% to fish growth [17]. The protein synthesis rate (Ks) can be determined as the percentage of an incorporated labeled tracer (e.g., phenylalanine) into proteins per day. High Ks values in white muscle have been found in juvenile fish (Ks = 4.4% day^−1^ [18]) and Ks values are low in fish from Antarctica (Ks = 0.04–0.2% day^−1^ [19,20,21]). Measuring the protein synthesis rate alone may already be indicative of growth performance, but in addition, protein degradation modifies net protein gain. Thus, combined assessment of protein synthesis and protein degradation will help to understand the temperature-dependent protein turnover for more accurate predictions of ectothermal (muscle) growth.

Protein degradation can be calculated as the difference of protein synthesis, food supply and weight gain [19,22]. While the protein degradation rates of fish from temperate waters were found to be higher at the upper end of their thermal window (*Lipophyrs pholis* acclimated for 28 days to minimum 3 °C and maximum 18 °C), fish from polar regions (*Harpagifer antarcticus*, acclimated for 28 days to between −1 °C and 3 °C) did not display any temperature-dependent changes in protein degradation [19]. Compared to protein synthesis, protein degradation is more complex and involves several pathways which may differ between species, life stages and tissue types [23]. Furthermore, the use of pathways can shift, e.g., with temperature as shown in the liver of the Antarctic eelpout, *Pachycara brachycephalum*, where warming induced a shift in protein degradation from the mostly ubiquitin-dependent pathway in the cold towards the lysosomal pathway in the warmth [14]. In contrast to fish liver and mammalian muscle tissue, degradation via the proteasome is less dominant in white muscle of fish, where the main degradation pathway is via the calcium-dependent protease calpain, closely followed by cathepsin (mainly cathepsin D), which is a lysosomal protease [23]. The maximum protein degradation rate is determined from the capacity of cathepsin D [24,25].

Both the measurements of protein synthesis and degradation quantify the use of a specific pathway. Another approach is non-targeted metabolic profiling which reflects metabolic differences or changes including in the levels of important amino acids, the consequences of shifts in energy budget and the fate of compounds related to protein synthesis and degradation. Metabolic profiling based on NMR (Nuclear magnetic resonance) spectroscopy has been applied to several organisms and results indicate that changes in protein synthesis and/or protein degradation are likely to occur under the influence of various environmental pressures (e.g., [26,27,28])).

Here we studied all three proxies to assess growth-related metabolic shifts during acute warming in the stenothermal Antarctic eelpout (*Pachycara brachycephalum*) and the eurythermal common eelpout (*Zoarces viviparus*). The two species provide an ideal model system to study evolutionary temperature adaptation due to their high genetic identity [29]. Both species belong to the family of Zoarcidae and most likely evolved in the North Pacific and reached the North Sea and Southern Ocean via the deep sea [30] and have evolved to divergent species-specific thermal windows [12,31,32]. The Antarctic eelpout inhabits the Southern Ocean at temperatures between −1 °C and 1 °C [33,34]. Besides very low temperature variation over the year, high seasonality of primary production is given by the extreme shift between permanent light and the Polar night. As the Antarctic eelpout is an opportunistic feeder of the benthos, high variation in food supply can be anticipated [30,33]. Their optimal temperature of growth was found to be between 3 and 4 °C in whole-animal [12,14] and cellular studies [35]. Antarctic eelpout can survive temperatures up to 12 °C in short-term experiments [36,37], but long-term acclimation indicates a tipping point for successful acclimation at around 6 °C [14]. This narrow thermal window indicates a low ability to adapt rapidly to thermal changes, and thus a low thermal plasticity, which can be defined as rapid adaptation to new thermal conditions.

In contrast, the distribution of the common eelpout ranges from the White Sea in the North, with winter temperatures as low as −1 °C, to the Wadden Sea in the South, at summer temperatures beyond 18 °C [38,39]. In short-term experiments *Z. viviparus* survived temperatures up to 24 °C and their thermal optimum (measured for the Wadden Sea population) ranges between 12 °C and 15 °C indicating a much wider thermal window and thus higher thermal plasticity [12,39,40]. Irrespective of species or habitat, studies of temperature-dependent whole-organism growth performance reveal a kind of bell-shaped curve [12,41,42,43,44,45]. Although growth is generally slow in Antarctic species, the recent literature suggested a lower thermal sensitivity of protein metabolism (synthesis and degradation rates) in Antarctic compared to temperate fish species when exposed to a similar range of warming [19].

In this context, a complete and comparative understanding of the relationship between protein synthesis and degradation rates as well as the associated metabolism in Antarctic and temperate fish is missing. In this study we hypothesize that acute warming affects protein synthesis and degradation rates in both eelpout species according to their thermal window: at the species-specific thermal growth optimum, protein synthesis rates should be high while protein degradation should be lowest.

## 2. Materials and Methods

### 2.1. Animals

Antarctic eelpout, *Pachycara brachycephalum* (Pappenheim, 1912), were caught in Admiralty Bay, King George Island (62°11′ S, 58°20′ W), Antarctica, by baited fish traps between 430 and 530 m depth on RV Polarstern expedition PS112 in March 2018. The fish traps were recovered after 52 h from the sea floor. On board, fish were kept at 0 °C for the duration of the transport (2 months) to the Alfred Wegener Institute (AWI), Bremerhaven, Germany. At AWI, fish were kept in well aerated, re-circulating seawater at 0.0 ± 0.5 °C and 34 ± 1 practical salinity units (PSU) under a 12:12 light/dark cycle.

The common eelpout, *Zoarces viviparus* (Linnaeus, 1758), were caught in the North Sea near the island of Helgoland and brought to AWI by RV Uthörn in autumn 2020. The fish were kept in well-aerated, re-circulating seawater at 12 °C for an acclimation period of at least 3 months before cooling the water slowly to a temperature of 4.0 ± 0.5 °C. For an additional three months, fish were maintained at 4 °C, at 34 ± 1 PSU on a 12:12 light/dark cycle.

Both species were fed frozen blue mussels, *Mytilus edulis* (Erdmann, Germany), 2–3 times weekly. Before the start of the experiments, fish were not fed for 5–7 days to minimize/exclude possible side effects like specific dynamic action (SDA). Handling and killing of the fish were conducted in compliance with the German legislation and in line with the recommendations of the American Veterinary Medical Association (AVMA). The work was approved by German authority (Freie Hansestadt Bremen, reference number 160; 500-427-103-7/2018-1-5).

### 2.2. Acute Warming Experiment

*P. brachycephalum* was acclimated to 0 °C and exposed to an acute temperature increase of +2 °C day^−1^ until 10 °C (total experimental duration: 5 days). The temperature of *Z. viviparus*, which was acclimated to 4 °C, was increased by +3 °C day^−1^ until at 22 °C (total experimental duration: 7 days). These acute warming experiments have been performed in other experiments with these fish and were therefore selected (e.g., [36,37]). At several temperature steps (*P. brachycephalum*: 0, 2, 4, 6, 8, and 10 °C; *Z. viviparus*: 4, 10, 13, 16, 22 °C) eelpouts of both species were weighed and injected with 0.7 mL/100 g body weight of 75 mM of ^13^C_9_H_11_^15^N_1_O_2_ phenylalanine in PBS buffer (pH 7.4). Exactly after 1.5 h and 3 h one fish from each species was sacrificed. At first the fish were stunned with a blow to the head and then killed by cutting the spinal cord behind the head before collecting muscle tissue. The collected tissue was flash-frozen in liquid nitrogen and stored at −80 °C until further use. This experiment was repeated four times for *P. brachycephalum* (total n = 47, for each measured temperature step n = 8 except of 10 °C n = 7) and three times for *Z. viviparus* (total n = 30, for each measured temperature step n = 6).

### 2.3. Measurement of Protein Synthesis Rate

The protein synthesis rate was measured as described in Krebs et al. [20]. Briefly, 50 mg of white muscle tissue was homogenized and extracted with methanol and chloroform to obtain three layers. The upper layer contained the cytosolic fraction in which the free pool of unlabeled and labeled phenylalanine was measured. The middle layer contained the protein fraction, which was subsequently hydrolyzed and used to measure the protein-bound fraction of labeled and unlabeled phenylalanine, and the bottom layer, containing the lipids, was not used for this analysis. Afterwards, the amount of free labeled and unlabeled phenylalanine as well as the amount of protein-bound labeled and unlabeled phenylalanine were measured with liquid chromatography high resolution mass spectrometry (LC-HRMS/MS).

The protein synthesis rate data were calculated with the phenylalanine concentrations in the protein hydrolysate (bound) and the cytosolic fraction (free pool) using an internal standard and a calibration curve for each analyte. Outliers (as identified by the Inter-Quartile Range IQR) were eliminated.

Ks was calculated after Garlick et al., 1980 [46]

$$\mathrm{Ks}\;(\%\;{\mathrm{day}}^{-1})=\left(\frac{Sb\;labeled[\frac{pg}{µg}]}{\left(Sb\;labeled+Sb\;unlabeled\right)[\frac{pg}{µg}]}\right)\times \left(\frac{100}{\left(Sa\;labeled\left[\%\right]\right)\times t(days)}\right)\times 100$$

where Sb is the protein-bound pool and Sa the free pool of phenylalanine (pg phenylalanine per µg fresh weight) and t the time in hours. Labeled phenylalanine describes the injected ^13^C_9_, ^15^N phenylalanine and unlabeled the naturally found ^12^C, ^14^N phenylalanine.

### 2.4. Protein Degradation via Measurements of Cathepsin D Activity

The determination of protein degradation and metabolic profiles was performed only at certain temperature steps, for *P. brachycephalum* at 0, 4 and 10 °C and for *Z. viviparus* at 4, 10, 13, 16 and 22 °C.

First, 50 mg of frozen white muscle tissue was homogenized (1 mg/100 µL) in 50 mM Sodium Acetate buffer (pH 5.0) in 2 circles of 20 s at 6000 rpm at 4 °C (Precellys 24 tissue homogenizer, Bertin Instruments). Afterwards the activity of cathepsin D was measured as described in Martinez-Alarcon et al., 2018. We used the fluorogenic substrate 7-methoxycoumarin-4-acetyl-Gly-Lys-Pro-Ile-Leu-Phe-Phe-Arg-Leu-Lys-(DNP)-DArg-amide (M0938, Sigma-Aldrich, St. Louis, MO, USA) and measured the activity of cathepsin D at 26 °C. Data are expressed in units per mg of protein (U mg^−1^), with protein content determined according to Bradford [47].

### 2.5. Metabolic Profiling

Metabolic profiling was conducted as described in detail in Tripp-Valdez et al. (2017) and Götze et al. (2020) [27,48]. In brief, we homogenized 50 mg muscle tissue (fresh weight) and extracted the cytosolic fraction by Methanol–Chloroform extraction. Afterwards, the cytosolic fraction was dried overnight in a vacuum centrifuge (Speedvac, Thermo Fisher Scientific, Waltham, MA, USA). The pellet was resolved with D_2_O (deuterized water + TSP; 0.075 wt%; Sigma Aldrich, St. Louis, MO, USA) in a 2-fold volume per fresh weight (final concentration: 0.5 g ml^−1^). For the determination of metabolites an ultra-shielded vertical 9.4 T NMR spectrometer (Avance III HD 400 WB, Bruker-BioSpin GmbH, Ettlingen, Germany) was used. Samples were transferred to NMR needle tubes (1.7 mm, Fisher Scientific, Schwerte, Germany) with a sample volume of 45 µL. ^1^H-NMR spectra were acquired at 400 MHz in a 1.7 mm triple-tuned ^1^H-^13^C-^15^N NMR probe using a Carr–Purcell–Meiboom–Gill (Bruker protocol cpmgpr1d, TopSpin 3.5) sequence including water suppression at room temperature at the following parameters: acquisition time (AQ), 4.01 s; sweep width (SW), 8802 Hz (22 ppm); delay (D1), 4 s; dummy scan (DS), 4; and number of scans (ns), 512.

Afterwards the spectra were baseline-, shim- and phase-corrected and calibrated to the TSP signal at 0.0 ppm using the software Chenomx NMR suite 8.4 (Chenomx Inc., Edmonton, AB, Canada). Then, the metabolites were assigned and quantified by the chemical shift of their NMR signals based on the TSP signal using Chenomx’s internal database and previous NMR studies on polar and marine fish [28,49].

### 2.6. Statistical Analyses

The data for protein synthesis and degradation rate were normally (tested with Shapiro–Wilk test) and homogeneously distributed (chi square test). Statistical differences at the level of 95% were tested by using an ordinary one-way ANOVA (analysis of variance) followed by the Tukey’s multiple comparison test as post hoc. Interspecific differences were tested with unpaired *t*-test identifying significant differences at the level of 95% confidence interval.

The metabolomic data were analyzed with the online platform MetaboAnalyst 5.0 [50]. First, the data were normalized using the log2 transformation and outliers were identified using unsupervised principal component analyses (PCA). Significant differences were investigated by using SAM (Significance Analyses of microarray) [51] and the distinction between metabolic profiles are presented by using supervised partial least-square discriminant analysis (PLS-DA).

## 3. Results

### 3.1. Protein Synthesis Rate

**Figure 1:**
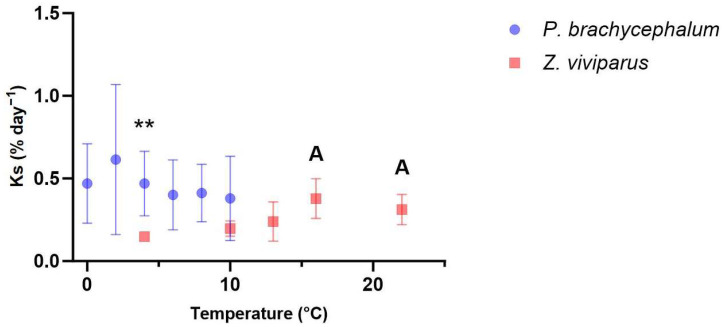
Protein synthesis rate in white muscle during acute warming. The protein synthesis rate in *P. brachycephalum* (blue, data shown as mean ± standard deviation) remained unchanged (One-Way ANOVA, *p* value > 0.05, n = 8 (2, 6 and 8 °C), n = 7 (0, 4, 10 °C)) during acute warming. In *Z. viviparus* (red, data shown as mean ± standard deviation), protein synthesis differed significantly at 16 and 22 °C from that at acclimation temperature of 4 °C (One-Way ANOVA, A = *p* value < 0.05, n = 6). The protein synthesis rate was significantly higher in the Antarctic than the North Sea eelpout at 4 °C but not at 10 °C (unpaired *t*-test ** *p* < 0.01).

Overall, the range of protein synthesis rates in white muscle tissue over all temperatures was higher in *P. brachycephalum* (Ks = 0.38–0.614% day^−1^) than in *Z. viviparus* (Ks = 0.148–0.379% day^−1^) with significantly higher Ks values in the Antarctic species at the common temperature of 4 °C (Figure 1). While the Ks of *P. brachycephalum* remained unchanged during acute warming despite high individual variation, the Ks of *Z. viviparus* displayed a clear temperature effect with the lowest protein synthesis rate at 4 °C (Ks = 0.148% ± 0.02 day^−1^) and the highest at 16 °C (Ks = 0.379% ± 0.12 day^−1^).

### 3.2. Protein Degradation

**Figure 2:**
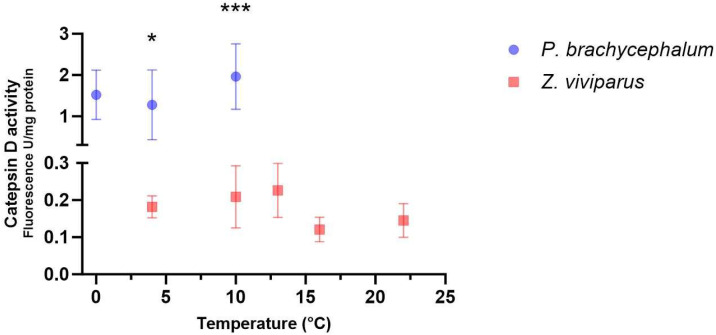
Protein degradation of *Pachycara brachycephalum* and *Zoarces viviparus* measured by the maximum activity of Cathepsin D at 26 °C (data shown as mean ± standard deviation). Acute warming of the animals did not significantly alter the activity of cathepsin D (One-Way ANOVA, *p* value > 0.05) in either *P. brachycephalum* (blue, n = 8 (4 °C), n = 7 (0 and 10 °C) or *Z. viviparus* (red, n = 6 (16 °C), n = 7 (4, 10, 13 and 22 °C). When measured at a common temperature (26 °C), Cathepsin D activity in white muscle samples collected at 4 and 10 °C was significantly higher in *P. brachycephalum* than in *Z. viviparus* (unpaired *T*-test; * *p* < 0.05; *** *p* < 0.005).

Protein degradation was determined in white muscle tissue by measuring the maximum activity of cathepsin D. The cathepsin D activity was 10 times higher in *P. brachycephalum* compared to *Z. viviparus* when measured at a common temperature (26 °C). When comparing white muscle tissue collected at different temperatures (*P. brachycephalum*: 0, 4 and 10 °C; *Z. viviparus*: 4, 10, 13, 16 and 22 °C), no significant effect on cathepsin D activity (all samples measured at 26 °C) was found in either species (Figure 2), indicating enzyme quantity remaining unchanged during the temperature protocols. Studies of homogenates at various temperatures revealed a Q_10_ for cathepsin activity of 2.3 ± 0.33 (n = 6) for *P. brachycephalum*. No difference was found in Q_10_ values between samples from various temperatures (0, 4 and 10 °C) (see Supplementary, Table S1).

### 3.3. Metabolites

A total of 47 metabolites mainly associated with protein turnover and energy allocation were assigned in white muscle of both species, *P. brachycephalum* and *Z. viviparus*. The metabolic response to acute warming differed between species and was more pronounced in *Z. viviparus* than in *P. brachycephalum*, as reflected in more metabolites changing significantly.

#### 3.3.1. *Pachycara brachycephalum*

**Figure 3:**
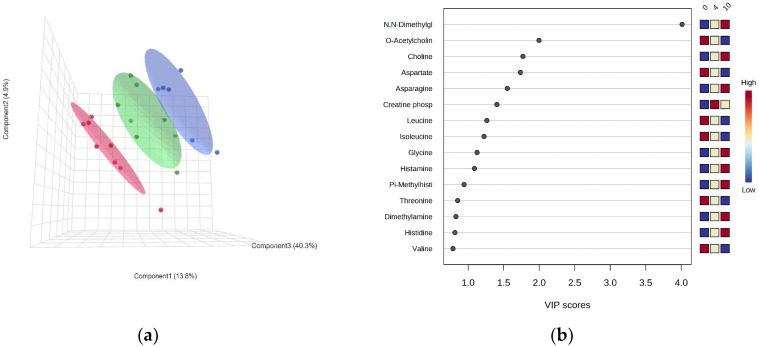
PLS-DA of the metabolic profile of *Pachycara brachycephalum*. The PLS-DA in a 3-component view contained 59% of the data (**a**) and the VIP score described the loadings for the metabolites most important for the description of the model (**b**). A clear separation between the 0 °C (red, n = 8), 4 °C (green, n = 8) and 10 °C (blue, n = 6) groups could be observed.

**Figure 4:**
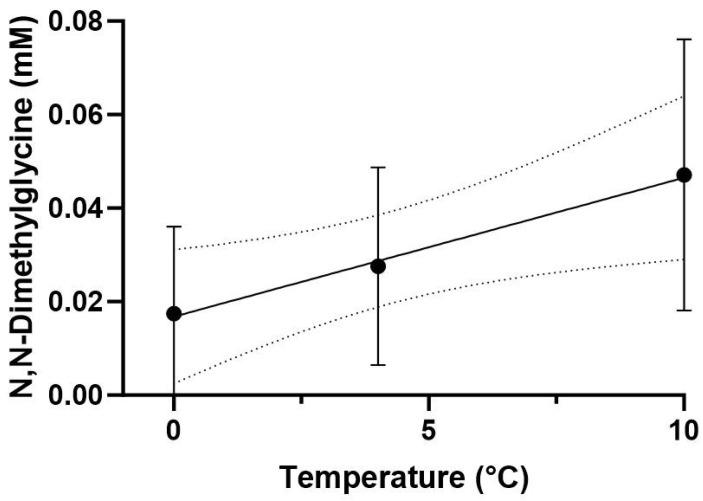
N,N-dimethylglycine level in *P. brachycephalum* during acute warming. The level of N,N-dimethylglycine increased linearly (Y = 0.002969x + 0.01681, R_2_ = 0.237, *p*-value < 0.05, data shown as mean ± standard deviation) with temperature (significant difference using Significance Analyses of Microarray SAM (Delta value 0.3, FDR 0.01, False 0.01, n = 8 (0 and 4 °C), n = 6 (10 °C)) with a *p*-value < 0.001.

The PLS-DA model indicated a clear separation of metabolic profiles in the white muscle of *P. brachycephalum* between the lowest (0 °C, red), intermediate (4 °C, green) and the highest (10 °C, blue) temperature (Figure 3a). Most importantly, the warming-induced increase in N,N-dimethylglycine levels mainly shaped the PLS-DA, followed by a decrease in acetylcholine levels and the increase in choline levels (Figure 3b) Additionally, mostly amino acid levels changed with warming, with increasing levels in, e.g., asparagine, glycine and histidine, and decreasing levels in, e.g., aspartate, leucine and isoleucine. Therefore, SAM revealed that N,N-dimethylglycine levels increased significantly and linearly with increasing temperature (Figure 4).

#### 3.3.2. *Zoarces viviparus*

**Figure 5:**
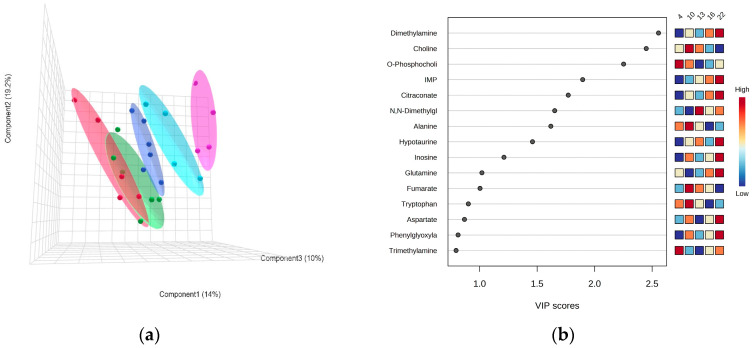
PLS-DA of the metabolite profile of *Zoarces viviparus*. The PLS-DA viewed in a 3D model included 43.2% of the data (**a**) and the VIP score described the loadings of the PLS-DA (**b**). There was no clear separation between values at 4 °C (red, n = 5) and at 10 °C (green, n = 6), but those at 13 °C (dark blue, n = 6), 16 °C (light blue, n = 4) and 22 °C (pink, n = 4) were clearly separated.

The PLS-DA model values for *Z. viviparus* did not show a clear separation at the lowest temperatures of 4 °C and 10 °C, but they were separated at higher temperatures of 13 °C (dark blue), 16 °C (light blue) and 22 °C (pink) (Figure 5a). Most importantly, warming resulted in an increased dimethylamine content (Figure S1), contributing to the PLS-DA patterns. Choline first increased and then decreased, forming a bell-shaped curve, while the phosphocholine content decreased with increasing temperature (Figure 5b).

**Figure 6:**
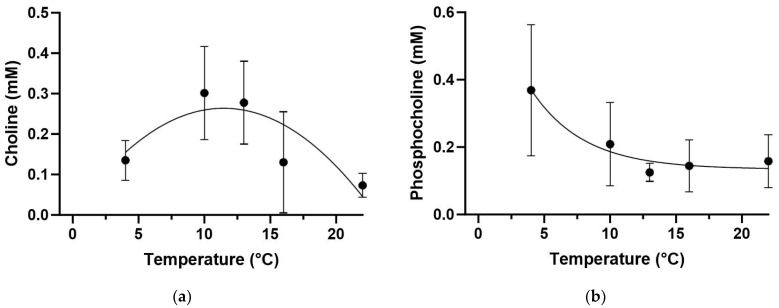
Changes in the concentration of choline and phosphocholine during acute warming in *Zoarces viviparus*. Choline (**a**) first increased and then decreased forming a bell-shaped curve (y = 0.0061x^2^ + 0.045x − 0.0020, R_2_ = 0.39, data shown as mean ± standard deviation) while phosphocholine (**b**) decreased (Y = 0.134 − (0.134 − 0.78)^(−0.25x)^, R_2_ = 0.39, data shown as mean ± standard deviation) during acute warming (significant difference using Significance Analyses of Microarray SAM (Delta value 0.5, FDR 0.188, False 0.46, (4 °C (n = 5), 10 °C (n = 6), 13 °C (n = 6), 16 °C (n = 4) and 22 °C (n = 4)) with a *p*-value < 0.001 (Choline) and *p* < 0.05 (Phosphocholine).

The concentration of choline and phosphocholine changed significantly with increasing temperature. Choline doubled its concentration between 4 and 10 °C and remained unchanged up to 13 °C. At higher temperatures the choline concentration decreased and formed a bell-shaped curve (Figure 6a). In contrast, the phosphocholine concentration decreased between 4 and 13 °C and reached a plateau at higher temperatures (Figure 6b).

#### 3.3.3. Comparison between the Antarctic and Common Eelpout

**Figure 7:**
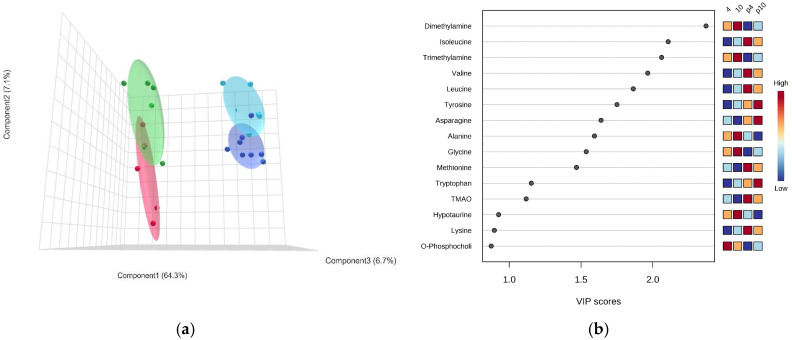
The PLS-DA of the metabolite profile of *P. brachycephalum* in comparison to *Z. viviparus* at 4 and 10 °C. The 3D model of the PLS-DA comprised 78% of the data (**a**) and the VIP score (**b**) describes the metabolites used for the PLS-DA of *P. brachycephalum* (dark blue 4 °C, n = 8; light blue 10 °C, n = 6) compared to *Z. viviparus* (red 4 °C, n = 5; green 10 °C, n = 6).

The metabolite profiles differed largely between the two species at the same temperatures (4 °C and 10 °C, Figure 7).

The PLS-DA indicated a clear separation between *P. brachycephalum* and *Z. viviparus* specimens at 4 and 10 °C (Figure 7a). The VIP score indicated that the main difference was found in the concentration of methylamines (dimethylamine, trimethylamine and TMAO (Trimethylamine-N-Oxide)) and amino acids (e.g., isoleucine, valine, leucine). While di- and trimethylamine were significantly higher in *Z. viviparus*, the concentration of TMAO was higher in *P. brachycephlaum*. Additionally, the concentration of most amino acids was significantly higher in *P. brachycephalum*, but those of alanine, histidine and glycine were significantly higher in *Z. viviparus* (Figure 7b). The SAM (Delta value 5.7, FDR 0.003, False 0.13, *p* < 0.005) identified 23 metabolites that differed significantly between the two eelpout species (Table S1). For better comparison, Table 1 summarizes the significant differences between *P. brachycephalum* and *Z. viviparus* as fold difference between the means at 4 and 10 °C for each species separately, as PLS-DA did not show a clear separation with respect to temperature (Table 1).

## 4. Discussion

In the following, thermal plasticity is discussed separately for both eelpout species, starting with *P. brachycpehalum* and then continuing with *Z. viviparus*. In the last part, the differences between the two species are interpreted to represent consequences of thermal adaptation.

### 4.1. Thermal Plasticity of the Cold-Stenothermal Pachycara brachycephalum 

The protein synthesis rate (Ks) in white muscle of *Pachycara brachycephalum* did not change during acute warming but varied highly between individuals at Ks = 0.5 +/− 0.25% day^−1^. Most strikingly, it was 10 times higher than in our previous *in vivo* study [20]. Compared to the former study, we increased the food supply from once a week [20] to 2–3 times a week in the present study, 3–6 weeks before the experiment started. In the Southern Ocean, food such as plankton is abundant in summer (December to March), while it decreases dramatically in winter (June to September) [52]. *P. brachycephalum* appears to be adapted to these drastic seasonal changes in food supply by up-regulating protein synthesis in white muscle when food is abundant and drastically reducing protein synthesis in white muscle when less food is available to conserve energy. Smith and Haschemeyer reported a reduction in the Ks by a factor of between 3 (*Trematomus bernacchii*) to 5.5 (*Trematomus hansoni*) in Antarctic fish due to a starvation period of 5 (*Trematomus bernacchii*) to 15 days (*Trematomus hansoni*) at −1.5 °C [21]. Although *P. brachycephalum* was not starved in our previous study, the higher feeding rate used here likely increased the Ks.

Acute warming did not affect the protein synthesis rate in white muscle of *P. brachycephalum*, resulting in a Q_10_ of about 1, far below a Q_10_ value commonly expected at 2–3. This contrasts expectations from long-term studies in thermally acclimated Antarctic organisms where growth below their thermal optimum follows a Q_10_ above 3 [6]. It is important to distinguish results obtained in acute warming experiments from those obtained after acclimation to different temperatures over weeks to months. To our knowledge, this is the first study to investigate protein synthesis rates *in vivo* in white muscle during acute warming in Antarctic fish. Our finding of protein synthesis being non-responsive to temperature is consistent with *in vitro* findings obtained from 3-months-acclimated *P. brachycephalum*, where the capacity for protein synthesis did not respond to temperature changes [32]. In fact, recent *in vivo* measurements on the Antarctic fish *Harpagifer antarcticus*, in which the protein synthesis rate was measured similarly, by use of a flooding dose of phenylalanine, did not reveal any change in the protein synthesis rate as a result of increased temperature, although the fish were acclimatized for 28 days at different temperatures and the protein synthesis rate was measured for the whole fish [19].

Our present results argue that the protein synthesis machinery remains unchanged after acute warming (+2 °C day^−1^) and operates at low Q_10_. Further experiments would need to clarify whether protein synthesis capacity is increased by protein expression during long-term cooling or reduced during long-term warming. This leaves the question open whether protein synthesis in white muscle can be thermally compensated in any Antarctic species.

Lysosomal degradation of proteins via cathepsin D did not differ significantly in white muscle samples at 0, 4 and 10 °C when measured at a common temperature (26 °C), indicating unchanged enzyme quantities. When cathepsin D activity was measured at different temperatures (∆T = 19 °C) the Q_10_ value averaged at 2.3 ± 0.33 (n = 6). Additionally, cathepsin D prefers a pH below 5 as found in the lysosome, even though it is also active at higher pH in extracellular space and cytoplasm [53]. In *P. brachycephalum*, acute warming has been shown to lower intracellular pH [37], which could further increase the cathepsin D activity during acute warming. It is therefore likely that lysosomal degradation *in vivo* was about 2.3 times lower at 0 °C than at 10 °C. This would need to be confirmed by *in vivo* studies, as many regulatory processes of cathepsin D are not yet fully understood, which could further influence cathepsin D activity *in vivo* [54].

To gain a deeper understanding of the metabolic changes, especially those involved in protein degradation, we performed untargeted metabolic profiling of white muscle tissue and identified 47 different metabolites at 0, 4 and 10 °C. A PLS-DA discriminant analysis shows a shift of metabolites towards warmer temperatures, with the metabolite N,N-dimethylglycine responding most strongly to elevated temperatures, as also confirmed by SAM. N,N-dimethylglycine is a derivate of glycine and an intermediate of choline metabolism. It enhances immune responses in salmonid fish [55] and prevents oxidative stress by scavenging free radicals that would otherwise damage cells, proteins and DNA [56]. N,N-dimethylglycine can be formed from choline via betaine to glycine; however, none of these three metabolites changed significantly during acute warming. In addition, PLS-DA revealed the neurotransmitter acetylcholine to decrease, and choline to increase, with acute warming. Acetylcholine is used by the nervous system as a transmitter to activate movement and can rapidly be converted to choline [57]. However, there is no evidence of increased protein degradation as the associated metabolites such as, e.g., 1-methylhistidine and 3-methylhistidine do not change with temperature (see supplementary, Figure S2).

In summary, acute warming has limited effects on *P. brachycephalum* white muscle as neither protein synthesis nor metabolites associated with protein degradation or the maximum protein degradation capacity changed. Similarly, protein degradation, calculated as the difference between protein synthesis, food supply and weight gain, did not change in the thermally acclimated (28 days) Antarctic fish *Harpagifer antarcticus* [19]. Both protein synthesis and protein degradation rates being thermally non-responsive in white muscle, points to other factors that may then lead to reduced growth rates at temperatures above the thermal optimum. For example, in a systemic to molecular hierarchy of thermal tolerance [58], energy-dependent protein synthesis and growth would increasingly be constrained according to OCLTT [1] while degradation might continue unabated. Clearly, further studies are necessary to clarify the limited effect of warming on Antarctic fish within its thermal range. In total, long-term temperature-dependent growths in the Antarctic eelpout [12,14] and other species cannot be explained from the present results of acute thermal changes.

### 4.2. Thermal Response of the Eurythermal Zoarces viviparus

In contrast to *P. brachycephalum*, the protein synthesis rate (Ks) in *Z. viviparus* white muscle increased with temperature to a maximum value of 0.38% day^−1^ at 16 °C resulting in a Q_10_ of about 2.2. At higher temperatures (22 °C), protein synthesis did not increase further but decreased slightly (Ks = 0.31% day^−1^). This suggests that, similar to *P. brachycephalum*, mechanisms such as OCLTT are involved to constrain the thermally induced increase in processes, to within the thermal window. Overall protein synthesis in white muscle responds to acute warming, supporting previously measured *in vivo* growth maxima between 12 and 15 °C [12,39,40].

As in *P. brachycephalum*, the maximum activity of cathepsin D measured at a common temperature (26 °C) of white muscle exposed to acute warming did not change, indicating that protein expression levels and thus enzyme quantities did not change. In *Z. viviparus* as in *P. brachycephalum,* a simple Q_10_ effect may have increased protein degradation via cathepsin at high temperatures. In fact, a study by Fraser et al. (2022), comparing an Antarctic and a temperate fish, found calculated rates of degradation in the temperate fish to increase towards the upper thermal limit after an acclimation period of 28 days similar to our acute warming approach [19]. Another important protein degradation pathway via calpain was not measured and may have been altered at high temperatures.

Untargeted metabolic profiling revealed several changes in cellular processes during acute warming in *Z. viviparus*. At lower temperatures (4 and 10 °C) the metabolic profile did not change, but at higher temperatures (13, 16 and 22 °C) the metabolic profiles separated the treatment groups from each other (Figure 5). The PLS-DA identified the key metabolites causing this difference to be dimethylamine, choline and phosphocholine as well as metabolites involved in energy production (e.g., IMP, citraconate, inosine and fumarate). The SAM revealed only two metabolites to be changed significantly, namely choline and phosphocholine.

The micronutrient and intermediate choline initially increased between 4 and 10 °C, then stabilized up to 13 °C and decreased thereafter, thereby closely following the long-term temperature-dependent growth curve of *Z. viviparus* [12,39,40]. In skeletal muscle, choline provides its methyl group as a micronutrient for several processes including protein and lipid metabolism as well as autophagy [59]. In contrast to choline, O-phosphocholine, which can be directly synthesized from choline by phosphorylation and is an important precursor of membrane lipids, was found highest in the cold (4 °C) and decreased with increasing temperature. High phosphocholine content was found to improve cold tolerance by increasing the ability to modify phospholipids in membranes to maintain their fluidity in cold conditions [60,61]. In agreement with our results, lipid analyses of acclimatized *Z. viviparus* indicated a high lipid content in the cold, especially for lipids important for membrane fluidity [62].

While all other metabolites did not differ significantly between treatments according to SAM, several metabolites of interest were identified in the PLS-DA indicating metabolic trends relating to energy metabolism. Increasing levels of citraconate could indicate an enhanced Krebs Cycle as it inhibits Cis-Aconitate from leaving the Krebs Cycle [63]. Slightly increasing glutamine and aspartate levels and decreasing alanine levels, as well as an increasing concentration of IMP, a degradation product of ATP, reflect increasing energy deficiency; however, future studies would need to confirm this interpretation. Amino acids of skeletal muscle proteins can be used to fuel other tissues such as the liver as shown for starving mammals [64] as well as migrating and spawning salmon [65,66].

In summary, in the North Sea eelpout, the increased energy demand of white-muscle protein synthesis is within the thermal performance curve, as indicated by minor changes of metabolites associated with energy production between 4 and 16 °C. The rate of protein synthesis in white muscle of *Z. viviparus* decreasing beyond 22 °C indicates limited energy supply to protein synthesis and growth, in line with the consequences of OCLTT at thermal limits (e.g., [1]). Experiments on gene expression during acute warming (0.08 °C min^−1^) of the eurythermal fish *Gillichthys mirabilis* support our findings, as genes related to the cell cycle and cell proliferation were reduced in the white muscle, possibly reflecting reduced energy expenditures [67].

### 4.3. Comparison between the Antarctic and Common Eelpout

When comparing *P. brachycephalum* from the Southern Ocean with closely related *Z. viviparus* from the North Sea at the same temperatures, potentially large differences in protein metabolism became apparent.

The protein synthesis rate was two to three times higher in *P. brachycephalum*, at the same temperatures and feeding rates, indicating cold-compensated *in vivo* activities at their lower optimum temperature. Even more striking, the capacity of the lysosomal protease cathepsin D, an indicator of protein degradation, was found to be more than ten times higher in *P. brachycephalum* compared to *Z. viviparus*, indicating a cold-compensated capacity in the Antarctic species. This also indicates a higher protein turnover in the Antarctic species compared to its temperate confamilial. Higher protein turnover is also indicated from the comparison of metabolic profiles as especially the complex amino acids including branched chain amino acids (leucine, isoleucine and valine) together with asparagine, methionine, tryptophan and lysine are found at significantly higher concentrations in the cytosol of *P. brachycephalum*. These amino acids, which include essential amino acids and are energetically expensive to synthesize, may preferably be recycled and not undergo final oxidation via the Krebs Cycle. Higher cytosolic concentrations as seen in the Antarctic eelpout may indeed enable a higher turnover of proteins. In contrast, glucogenic amino acids like glycine and alanine, which can be synthesized quite simply from the glucogenic pathways, were found at much higher levels in *Z. viviparus*. Storch et al. (2005) found higher concentrations of glycine bound in proteins in *Z. viviparus* compared to *P. brachycephalum* [32], which may be a consequence of its higher abundance in the cytosol. Alanine is an important nitrogen carrier and a major gluconeogenic precursor in fish [68]. Depending on the metabolic state, it is released from skeletal muscle and often a precursor of other non-essential amino acids [69]. Together, these amino acids and their high concentrations may indicate a higher metabolic turnover and a different use of fuels in the two eelpouts, as described earlier [12].

The only essential amino acid found at higher concentration in the temperate eelpout is histidine, but the factorial change is quite low in comparison to the other complex amino acids. The histidine concentration acts as a buffer against changing pH values. For example, it increased in white muscle in salmon before migration to prepare them for the intense exercise [68,70]. Higher histidine concentrations in the temperate eelpout may thus be helpful in the more variable environment of the North Sea including temperature, oxygen and salinity. Overall, the differences in cytosolic amino acids between *Z. viviparus* and *P. brachycephalum* may reflect differences in environmental variability. *Z. viviparus* has a high proportion of simple amino acids that can be metabolized quickly under more variable conditions. In contrast, *P. brachycephalum* experiences stable temperatures and almost no seasonal variation, but food scarcity may occur. When a high level of food is available, it seems to respond quickly by increasing protein turnover and enhancing growth (as discussed above).

Alternatively, the increased protein turnover rates in Antarctic fish may be due to proteins being less stable in the cold [71], as a consequence of structural flexibility [72]. Several Antarctic fish species possess high levels of ubiquitin-conjugated proteins, suggesting a higher rate of degradation [73]. The hypothesis of higher protein instability would be supported by high level of TMAO in *P. brachycephalum*, while trimethylamine and dimethylamine are higher in *Z. viviparus*. TMAO is an osmolyte important for protein stabilization in marine organisms at depth [74]. *P. brachycephalum* can be found at much greater depth than *Z. viviparus* from the shallow North Sea. Whether TMAO is higher in polar organisms for stabilizing proteins in the cold needs to be investigated in the future.

## 5. Conclusions

The two eelpout species from the North Sea and the Southern Ocean respond differently to acute warming. The protein synthesis rate in the common eelpout *Zoarces viviparus* was increased between 4 and 16 °C according to the Q_10_ rule, a phenomenon not observed in the Antarctic eelpout. At 22 °C, the protein synthesis rate in white muscle was reduced in the temperate species. As protein synthesis is the largest energy consumer in ectotherms [75] our earlier findings [39] indicate that whole-animal constraints, e.g., through OCLTT, may have set in at upper thermal limits and reduced white-muscle growth. Within the thermal range of the Antarctic eelpout *Pachycara brachycephalum*, the rate of protein synthesis did not respond to acute warming. The increased protein synthesis rate, possibly due to an increased feeding rate (10-fold higher than in our previous study [20], might have masked the thermal response.

Regardless of temperature, protein turnover is significantly higher in *P. brachycephalum* compared to *Z. viviparus* despite the same feeding rate. This indicates cold compensation in *P. brachycephalum*, which can maintain higher metabolic rates than *Z. viviparus* at temperatures around 0 °C. Cold compensation is defined as “the maintenance of an appropriate physiological rate in the face of temperature change” [8]. While protein synthesis and degradation are cold-compensated, other functions such as reproduction and development may appear suppressed in order to enable metabolic down-regulation (cf. [7]). The present study indicates clear cold compensation of both protein synthesis rate and degradation in Antarctic fish. The functional background of this compensation needs further attention.

## Figures and Tables

**Table 1:** Overview of the different levels of metabolites in *Zoarces viviparus* and *Pachycara brachycephalum*: Fold differences between *P. brachycephalum* (PB) and *Z. viviparus* (ZV) were calculated using mean values at 4 and 10 °C separately for each species. Metabolites not significantly different according to SAM (see Supplementary, Table S2) or with fold differences below 2, and metabolites with values changing within species between 4 and 10 °C, were not considered. Bold numbers indicate a significantly higher concentration of the studied metabolite.

Metabolite (Name)	Concentration in *Z. viviparus* (mM)	Concentration in *P. brachycephalum* (mM)	Fold Difference of Means between PB and ZV
Amino acids			
Isoleucine	0.08 ± 0.04	**0.69 ± 0.34**	9.1
Valine	0.14 ± 0.07	**1.11 ± 0.55**	8
Leucine	0.12 ± 0.05	**0.90 ± 0.43**	7.2
Asparagine	0.11 ± 0.06	**0.56 ± 0.20**	5.6
Methionine	0.07 ± 0.03	**0.31 ± 0.14**	4.6
Tryptophan	0.03 ± 0.01	**0.11 ± 0.04**	3.4
Lysine	0.67 ± 0.26	**1.62 ± 0.59**	2.4
Glycine	**14.59 ± 5.43**	2.43 ± 0.78	0.17
Alanine	**5.96 ± 1.30**	1.11 ± 0.34	0.19
Histidine	**0.86 ± 0.32**	0.38 ± 0.14	0.44
Methylamine			
TMAO	7.54 ± 4.62	**23.15 ± 3.49**	3.1
Dimethylamine	**1.93 ± 1.32**	0.09 ± 0.05	0.05
Trimethylamine	**0.21 ± 0.13**	0.014 ± 0.003	0.07
Others			
Hypotaurine	**0.002 ± 0.001**	0.0007 ± 0.0003	0.35
Taurine	**8.96 ± 2.42**	3.94 ± 0.92	0.44

## Data Availability

Data will be uploaded to the public repository PANGEA after acceptance of the manuscript.

## Supplementary Materials

The following supporting information can be downloaded at: https://www.mdpi.com/article/10.3390/biom13101507/s1, Table S1: Q_10_ values of cathepsin D activity in muscle homogenates at various temperatures in *P. brachycephalum*; Table S2: SAM analysis comparison between metabolic profiles of *P. brachycephalum* and *Z. viviparus*; Figure S1: Changes of the concentration of dimethylamine during acute warming in *Zoarces viviparus*; Figure S2: Changes of the concentration of 1-Methylhistidine and 3-Methylhistidine during acute warming in *Pachycara brachycephalum*.
