# Supplementary material for: Evolutionary Adaptation of Protein Turnover in White Muscle of Stenothermal Antarctic Fish: Elevated Cold Compensation at Reduced Thermal Responsiveness
Source: Biomolecules. 2023 Oct 11;13(10):1507. doi: 10.3390/biom13101507 (PMC10605280; doi:10.3390/biom13101507)
Supplement: Supplementary file 1 [file biomolecules-13-01507-s001.zip › biomolecules-2649545-supplementary.pdf]

Supplementary Materials:

Table S1. Q_{10} values of cathepsin D activity in muscle homogenates at various temperatures in *P. brachycephalum*. For the Q_{10} calculation, the slope of cathepsin D activity at several temperature steps (ΔT = 19 °C) was determined for samples from 6 individuals at different temperatures (0, 4, and 10 °C).

Acclimation Temperature	Q_{10}
0°C	2.54
0°C	2.88
4°C	1.95
4°C	2.06
10°C	2.15
10°C	2.05
Average	2.27 ± 0.33

Table S2. SAM analysis comparison between metabolic profiles of *P. brachycephalum* and *Z. viviparus*. It describes the results of the SAM (Delta value 5.7, FDR 0.003, False 0.13) comparing *P. brachycephalum* and *Z. viviparus* at 4°C and 10°C.

Metabolite	d.value	stdev	rawp	q.value
Alanine	54.298	0.020949	0	0
Asparagine	34.678	0.0344	0	0
Glycine	34.124	0.035371	0	0
Tyrosine	29.339	0.039696	0	0
Trimethylamine	27.177	0.083969	0	0
Tryptophan	26.941	0.020915	0	0
Leucine	26.763	0.054808	0	0
Isoleucine	26.728	0.069668	0	0
Valine	24.481	0.065498	0	0
Choline	22.99	0.017189	0	0
Taurine	22.532	0.01147	0	0
Dimethylamine	17.399	0.17601	0	0
TMAO	15.119	0.049399	0	0
Serine	13.897	0.0079914	0	0
Glutamine	13.67	0.0074317	0	0
O-Phosphocholine	13.307	0.027079	0	0
Methionine	12.91	0.076114	0	0
Lysine	9.4739	0.034614	0	0
Hypotaurine	8.3763	0.045886	0.00022222	0.00025571
O-Acetylcholine	7.7886	0.024056	0.00044444	0.00044169
Fumarate	7.5165	0.012557	0.00044444	0.00044169
Histamine	7.4313	0.025264	0.00044444	0.00044169
Histidine	6.6808	0.043659	0.0028889	0.0025265

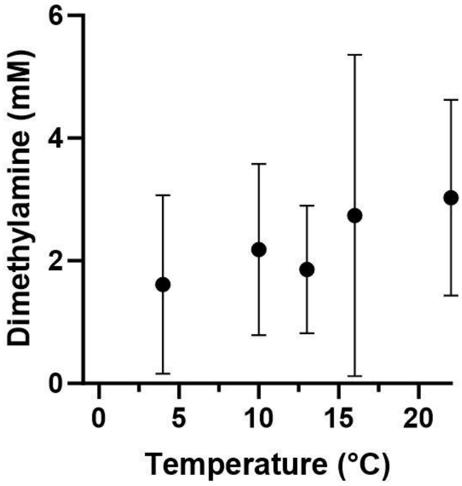

Figure S1. Changes of the concentration of dimethylamine during acute warming in *Zoarces viviparus*. Dimethylamine is not increasing significantly (significant difference using Significance Analyses of Microarray SAM (Delta value 0.5, FDR 0.188, False 0.46, (4 °C (n = 5), 10 °C (n = 6), 13 °C (n = 6), 16 °C (n = 4) and 22 °C (n = 4)).

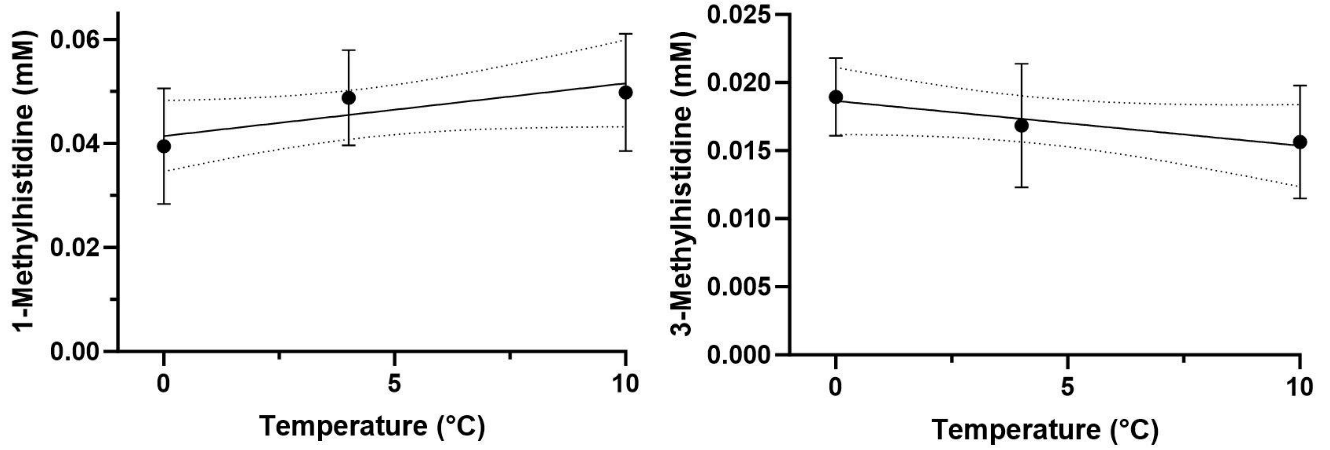

(**a**) (**b**)

Figure S2. Changes of the concentration of 1-Methylhistidine and 3-Methylhistidine during acute warming in *Pachycara brachycephalum*. Neither 1-Methylhistidine (Y = 0,001013x + 0,04142, R_2= 0.137, p-value < 0.05) nor 3-Methylhistidine (Y = -0,0003283x + 0,01865, R_2= 0.113, p-value < 0.05) changed significantly during acute warming.
